# Tooth Loss and Cognitive Performance Across Multiple Domains in Community‐Dwelling Older Adults in the United States

**DOI:** 10.1155/jare/8348807

**Published:** 2026-11-09

**Authors:** Huabin Luo, Xiang Qi, Zhijing Xu, Ruotong Liu, Yunrui Liu, Bei Wu

**Affiliations:** ^1^ Brody School of Medicine East Carolina University Greenville 27834 North Carolina USA ecu.edu; ^2^ Rory Meyers College of Nursing New York University New York 10010 New York USA nyu.edu; ^3^ NYU Shanghai Global Institute for Longevity and Resilience Shanghai 200124 China

**Keywords:** cognition, dental hygiene, dental visits, gerontology, oral health

## Abstract

**Background:**

Growing evidence suggests that poor oral health, particularly tooth loss, is associated with cognitive decline. This study examined the association between tooth loss and global and domain‐specific cognitive performance and evaluated how dental care utilization and oral hygiene practices influenced these associations among community‐dwelling older adults.

**Methods:**

Data were obtained from the National Health and Nutrition Examination Survey. The sample included 2099 adults aged ≥ 60 years with at least one natural tooth. Cognitive performance was assessed using standardized assessments of global cognition, word memory, verbal fluency, and attention and processing speed. Significant tooth loss was defined as the loss of ≥ 8 permanent teeth. Dental visits, dental flossing, and mouthwash use were self‐reported. Survey‐weighted logistic regression models were used to examine the associations.

**Results:**

Significant tooth loss was associated with lower cognitive performance in crude analyses and remained associated with lower verbal fluency after adjustment for sociodemographic and health‐related factors. Further adjustment for dental care utilization and oral hygiene behaviors resulted in additional attenuation of the association between tooth loss and cognitive performance. Daily flossing was independently associated with lower odds of global low cognition (AOR = 0.51, 95% CI: 0.28–0.91), low verbal fluency (AOR = 0.66, 95% CI: 0.47–0.93), and low word memory (AOR = 0.69, 95% CI: 0.49–0.98).

**Conclusions:**

Associations between tooth loss and poorer cognitive performance were attenuated after accounting for dental care and oral hygiene behaviors. Dental flossing was associated with better performance on several cognitive outcomes, although patterns varied across measures.

## 1. Introduction

As the U.S. population ages, the number of individuals living with dementia is expected to rise significantly. Currently, an estimated 6.9 million Americans aged 65 or older are living with Alzheimer’s dementia. This number may grow to 13.8 million by 2060 [1]. This alarming trend underscores the urgent need to identify modifiable factors of dementia and enhance prevention [2].

Growing evidence suggests that poor oral health—particularly tooth loss and periodontal disease—is associated with cognitive impairment and dementia [3–9]. Periodontal disease, which affects approximately three quarters of older adults [10] is a leading cause of tooth loss [11]. Maintaining oral health requires regular access to dental care and adherence to effective oral hygiene practices. Access to dental services enables early diagnosis, prevention, and clinical treatment of oral diseases such as caries and periodontitis. Likewise, daily oral hygiene behaviors, such as toothbrushing and flossing, are essential for preserving oral health and may be associated with better cognitive function.

Several studies have found that regular dental visits were associated with slower rates of cognitive decline [12, 13], while better oral hygiene practices have been associated with a reduced risk of cognitive impairment and dementia [13–15]. However, much of the existing research has focused on cognitive impairment or diagnosed dementia, with less attention paid to earlier stages of cognitive decline, including mild cognitive impairment, or to lower cognitive performance before clinically diagnosed dementia. Furthermore, few studies have examined dental care utilization and oral hygiene practices in relation to the observed association between tooth loss and cognitive performance.

Understanding the relationship between tooth loss, dental care utilization, oral hygiene practices, and cognitive performance may help inform strategies for promoting cognitive health among older adults. This study aimed to examine how dental care utilization and oral hygiene practices influenced the observed association between tooth loss and cognitive performance—including both global cognition and specific cognitive domains—among community‐dwelling older adults. We hypothesized that the observed association between tooth loss and cognitive performance would be attenuated after adjustment for dental care utilization and oral hygiene practices.

Figure 1 presents the conceptual framework underlying this study. The framework illustrates the hypothesized relationships among tooth loss, dental care utilization, oral hygiene practices, cognitive performance, and potential confounding factors. It summarizes the conceptual relationships examined in this study and is not intended to depict causal pathways.

**FIGURE 1:**
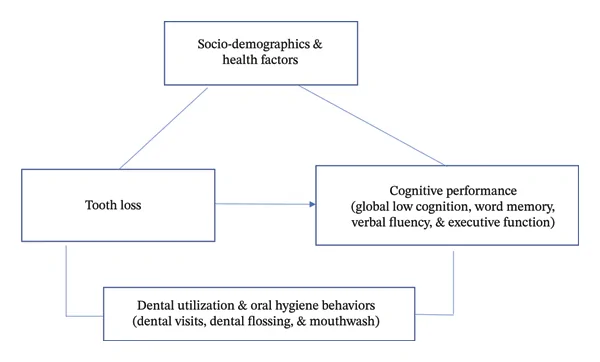
Conceptual framework of the association between tooth loss and cognitive performance.

## 2. Materials and Methods

### 2.1. Data Source

Data for this cross‐sectional analysis were from the 2011–2012 and 2013–2014 U.S. National Health and Nutrition Examination Survey (NHANES), which are the most recent NHANES waves that included cognitive function assessments. The cognitive tests were administered only in adults aged 60 years or older. NHANES is a stratified multistage probability sample of the civilian noninstitutionalized population in the United States. It includes interviews and physical and oral health examinations. The interviews include questions on sociodemographics, chronic conditions, dental care utilization, and oral hygiene practices. The examination protocol consists of physical, dental, and laboratory tests administered by highly trained medical personnel in a mobile examination center [16].

In this analysis, participants with missing data on cognitive tests (*n* = 698) were excluded. In addition, participants with missing data on tooth count (*n* = 192) were excluded. Given the study design, participants who had lost all 28 permanent teeth (*n* = 439) were also excluded because edentulous individuals may have substantial differences in dental visits compared with dentate individuals [17], and interdental cleaning behaviors such as dental flossing are not directly applicable to individuals without natural teeth. Finally, participants with missing data on covariates (*n* = 204) were excluded (see Figure 2 sample selection flow chart). The final analytical sample included 2099 participants aged 60 years or older.

**FIGURE 2:**
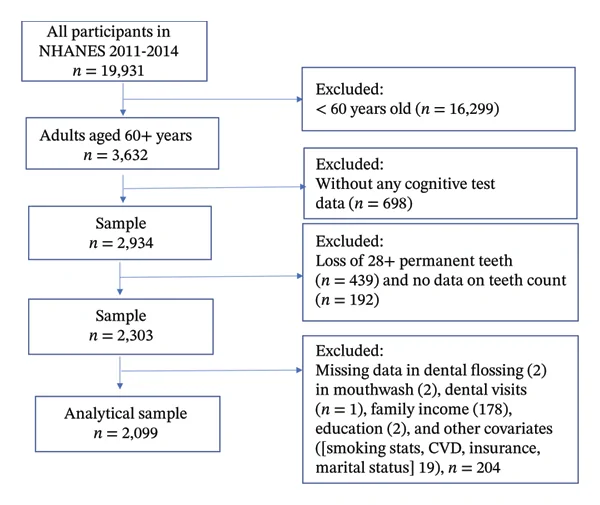
Flowchart of sample selection.

### 2.2. Measures

#### 2.2.1. Outcomes

In NHANES 2011–2014, cognitive performance was assessed using three validated cognitive assessments: (a) the Consortium to Establish a Registry for Alzheimer’s Disease (CERAD) battery was used to assess immediate verbal learning (CERAD‐WL) and delayed verbal memory (CERAD‐DR) [18]. The CERAD‐WL involves word learning and immediately recalling a list of 10 words across three trials (scores ranging 0–30); the CERAD‐DR requires recalling the word list after a short delay (scores ranging 0–10). (b) the Animal Fluency Test (AFT) was used to examine verbal fluency [19]. Participants were asked to name as many animals as possible in 1 min, with the score equal to the total named (scores ranging 0–40). (c) the Digit Symbol Substitution Test (DSST) was used to evaluate attention and processing speed [20]. Participants were asked to match symbols to numbers 1–9 under time pressure (2 min), with a maximum possible score of 133 (i.e., total number of correct symbols drawn). Participants who were unable to complete the practice items for the AFT or DSST did not proceed to the full test. Higher scores reflect better cognitive performance on all assessments. The outcomes included global cognitive performance and performance in specific cognitive domains.

*Global cognitive performance*. Because educational attainment strongly influences cognitive test performance, participants were first stratified into three education levels: less than high school, high school, and some college or above, consistent with prior research [21, 22]. Then, within each education stratum, a standardized score was calculated for each of the four cognitive tests. Third, the four standardized scores were averaged to create a global cognitive *z* score. Global low cognition was defined as a global cognitive *z* score at least one standard deviation below the stratum‐specific mean.

*Low performance in specific cognitive domains*. We defined binary indicators for low word memory, low verbal fluency, and low attention and processing speed based on established criteria: the cutoff for low memory was below 22 (out of a total of 40 of WL and DR) [23], below 14 for low AFT [24, 25], and below 34 for DSST [26, 27].

#### 2.2.2. Independent Variable

The primary independent variable was tooth loss. During the dental examination, the total number of missing teeth (excluding third molars) was recorded for each participant (ranging from 0 to 28). We operationalized tooth loss as a categorical variable: no missing teeth, nonsignificant tooth loss, and significant tooth loss (STL). STL was defined as a loss of eight or more teeth, and non‐STL was defined as a loss of 1–7 teeth [28].

### 2.3. Covariates

#### 2.3.1. Dental Use and Oral Hygiene Behaviors

*Dental visits*. Respondents were asked when they visited a dentist: 6 months or less, 6 months but less than 1 year ago, 1 year but not more than 2 years, 2 years but not more than 3 years, 3 years but not more than 5 years, more than 5 years, and never. For analysis, we dichotomized this variable as having visited a dentist within the past 12 months (yes) versus not in the past 12 months (no) [29]. *Mouthwash use*. Participants reported the number of days (ranging from 0 to 7 days) they used mouthwash for dental problems in the last 7 days. We converted it to a four‐category variable: 0 days (no mouthwash), 1–3 days (occasional mouthwash), 4–6 days (frequent mouthwash), and 7 days (daily mouthwash). *Dental flossing*. Similarly, participants reported the number of days (ranging from 0 to 7 days) they used dental floss or an interdental cleaning device in the last 7 days. We coded it as a four‐category variable: 0 days (no flossing), 1–3 days (occasional flossing), 4–6 days (frequent flossing), and 7 days (daily flossing).

#### 2.3.2. Sociodemographic, Chronic Condition, and Smoking Status Variables

Sociodemographic variables included age (60–64, 65–74, 75–79, and 80+), sex (male or female), self‐reported race (non‐Hispanic white, non‐Hispanic black, Hispanic [Mexican Americans or other Hispanic Americans], and other [including multiracial]), marital status (married or living with a partner vs. other), and educational level (less than high school, high school or a GED, or some college and above), family income (i.e., the ratio of family income to the federal poverty level [FPL] multiplied by 100: < 133%, 133%–< 350%, and ≥ 350%), and health insurance coverage (yes/no). *Chronic conditions* included high blood pressure (yes/no), diabetes (yes/no), cardiovascular disease (CVD) (yes/no, including congestive heart failure, coronary heart disease, angina, heart attack, or stroke). *Cigarette smoking status* (current smokers, former smokers, never smoked) was also included.

### 2.4. Statistical Analysis

We used survey‐weighted bivariate and multivariate procedures in data analysis to account for the complex survey design of NHANES. First, we assessed bivariate associations between participant characteristics and low cognitive performance (i.e., global low cognition, low word memory, low AFT, and low DSST). Then, we fitted a series of nested logistic regression models to examine how adjustment for dental care utilization and oral hygiene behaviors influenced the observed association between tooth loss and cognitive performance: Model 1 was the unadjusted (crude) model; Model 2 adjusted for sociodemographic variables, chronic diseases, and smoking status; and Model 3 further adjusted for dental visits, mouthwash use, and dental flossing. As an additional analysis, we evaluated whether the associations between tooth loss and cognitive performance differed according to dental care utilization and oral hygiene behaviors by including interaction terms between tooth loss and each dental behavior variable in the fully adjusted models. Data analysis was performed using Stata 16 (Stata Corp LP). A significance level of 0.05 was used.

## 3. Results

Table 1 presents participants’ characteristics. Briefly, about 25.5% of participants lost eight or more teeth (i.e., STL); 23.2% did not visit a dentist in the past 12 months; 46.2% did not use mouthwash in the last 7 days; and 24.1% did not use dental floss in the last 7 days.

**TABLE 1:** Respondent characteristics by cognitive status.

Variables	Sample		Global low cognition		Low word memory		Low AFT		Low DSST	
	*n*	%	%	*p*	%	*p*	%	*p*	%	*p*
Missing teeth				< 0.001		< 0.001		< 0.001		< 0.001
None	311	23.0	3.5		14.1		8.8		3.4	
1–7	1062	51.5	6.1		18.8		18.5		9.3	
8–27 (significant tooth loss)	726	25.5	12.3		32.0		31.1		24.4	
Age groups				< 0.001		< 0.001		< 0.001		< 0.001
60–64	707	34.5	3.0		12.5		13.7		6.3	
65–74	878	42.8	4.9		18.0		18.2		10.2	
75–79	200	9.5	10.8		34.2		24.6		20.5	
80+	314	13.2	25.3		45.2		37.5		31.2	
Sex				0.83				0.54		0.66
Male	1026	46.7	7.5		25.4		19.3		12.4	
Female	1073	53.3	7.7		18.1		20.5		12.9	
Race/ethnicity				0.26		< 0.001		< 0.001		< 0.001
Non‐Hispanic White	1029	80.9	7.4		19.7		16.4		8.4	
Non‐Hispanic Black	489	7.7	10.0		26.5		38.2		32.6	
Hispanic	389	6.6	8.7		36.0		30.1		40.3	
Other	192	4.8	10.1		22.2		42.4		13.0	
Married				0.22		0.01		0.01		< 0.001
No	839	32.9	10.4		25.0		25.0		19.8	
Yes	1260	67.1	6.3		19.6		19.6		9.1	
Education level			NA			< 0.001		< 0.001		< 0.001
< High school	432	12.0			43.7		40.7		42.6	
High school	462	20.4			25.4		26.8		15.2	
Some college or above	1205	67.6			15.8		13.7		6.1	
Family income level				< 0.001		< 0.001		< 0.001	0.0	< 0.001
< 130% FPL	551	15.0	12.8		40.3		33.8		32.2	
131%–350% FPL	817	38.2	9.4		24.3		22.6		14.7	
≥ 350% FPL	731	46.8	4.6		13.1		12.3		3.9	
Insurance				0.33		0.91		0.83	0.0	0.01
No	167	5.2	5.2		20.8		20.9		21.3	
Yes	1932	94.8	7.8		21.4		19.9		12.2	
High blood pressure				< 0.001		0.01				< 0.001
No	815	41.8	5.3		17.7		16.3		8.5	
Yes	1284	58.2	9.2		23.9		22.6		15.7	
CVD				< 0.001				0.01		< 0.001
No	1729	82.6	6.6		19.0		18.2		10.9	
Yes	370	17.4	12.7		32.9		27.6		21.0	
Diabetes				0.03		0.08			0.0	< 0.001
No	1631	81.4	7.0		20.6		18.4		11.2	
Yes	468	18.6	11.0		24.7		26.8		19.1	
Cigarettes smoking				0.20		0.48		0.87		0.27
Current smokers	225	9.3	7.5		23.3		21.4		15.7	
Former smokers	785	38.7	6.3		20.0		19.6		11.8	
Never smoked	1089	52.0	8.7		22.1		20.0		12.8	
Dental visit in the past year				< 0.001		< 0.001		< 0.001	0.0	< 0.001
No	675	23.2	10.7		29.6		28.8		24.4	
Yes	1424	76.8	6.6		18.8		17.1		9.0	
Mouthwash in the last 7 days				0.18		0.61		0.20		0.08
0	861	46.2	6.61		20.7		17.8		9.5	
1–3	321	16.2	9.37		22.9		18.9		13.1	
4–6	122	6.7	3.4		16.7		16.4		11.7	
7	795	30.9	8.19		22.5		22.4		14.2	
Dental flossing in the last 7 days				< 0.001		< 0.001		< 0.001		< 0.001
0	640	24.1	13.0		31.5		30.7		23.0	
1–3	439	23.9	4.39		17.7		15.3		7.0	
4–6	174	8.9	7.52		21.5		9.2		7.2	
7	846	43.1	5.77		17.7		17.2		9.0	

*Note:* %: weighted. The low cognition status was stratified by education level. CVD: cardiovascular disease.

Abbreviation: FPL: federal poverty level.

Participants aged 80 years or older had the highest proportion of lower cognitive performance (i.e., global low cognition, low word memory, low AFT, and low DSST). The proportions of lower cognitive performance were the largest in those with the lowest family income and lowest education levels. The proportions of lower cognitive performance were larger in those with chronic conditions (i.e., high blood pressure, diabetes, or CVD). Last, the proportions were also larger in those who did not have a dental visit in the past 12 months or did not use dental floss (all *p*s < 0.001, Table 1).

About 7.3% (95% CI: 6.1–8.8) of participants had global low cognition, 21.4% (95% CI: 19.0–24.0) had low word memory, 19.3% (95% CI: 17.6–21.0) had low AFT, and 11.7% (95% CI: 9.8–14.0) had low DSST (data not shown in table).

Table 2 presents the crude and adjusted odds ratios from logistic regression models for the outcome variable—global low cognition. In Model 1 (the crude model), the crude odds ratio for STL was significant (OR = 3.81, 95% CI: 2.15–6.71), indicating that STL was associated with higher odds of global low cognition; in Model 2, adjusting for sociodemographic variables, chronic conditions, and smoking status, the magnitude of the association was reduced (AOR = 1.77, *p* = 0.06). However, in Model 3, where the three dental care variables—dental visits, mouthwash, and dental flossing—were further controlled, the association between STL and global low cognition was attenuated and no longer statistically significant (AOR = 1.48, *p* = 0.19). Dental flossing (*p* = 0.02) was associated with lower odds of global low cognition. For instance, daily flossing (i.e., flossing every day in the last 7 days) was associated with lower odds of global low cognition (AOR = 0.51, 95% CI: 0.28–0.91). That is, in comparison with those who did not use dental floss, participants who flossed daily had 49% lower odds of having global low cognition. Neither having a recent dental visit (*p* = 0.73) nor using mouthwash (*p* = 0.36) was significantly associated with global low cognition in the fully adjusted model (Model 3).

**TABLE 2:** Logistic regression model results of factors associated with global low cognition.

Variables	OR	95% CI		*p*	AOR	95% CI		*p*	AOR	95% CI		*p*
	Model 1				Model 2				Model 3			
Missing teeth (vs. 0 missing teeth)				< 0.001				0.07				0.12
1–7	1.71	0.93	3.15	0.08	0.96	0.54	1.72	0.89	0.97	0.55	1.71	0.90
8–27	3.81	2.15	6.77	< 0.001	1.77	0.98	3.19	0.06	1.48	0.81	2.69	0.19
Age groups (vs. 60–64)												
65–74					1.61	0.74	3.52	0.22	1.66	0.76	3.63	0.19
75–79					4.41	1.73	11.22	< 0.001	4.60	1.79	11.78	< 0.001
80+					10.84	3.92	29.94	< 0.001	11.28	4.08	31.21	< 0.001
Female					0.81	0.51	1.28	0.35	0.86	0.53	1.40	0.54
Race/ethnicity (vs. non‐Hispanic white)												
Non‐Hispanic black					1.28	0.69	2.38	0.42	1.16	0.63	2.14	0.63
Hispanic					1.13	0.66	1.94	0.64	1.01	0.58	1.75	0.97
Other					1.12	0.49	2.58	0.78	1.24	0.54	2.82	0.60
Married					1.09	0.69	1.72	0.70	1.10	0.69	1.76	0.68
Family income (< 131% FPL)												
131%–350% FPL					0.76	0.43	1.34	0.33	0.79	0.43	1.44	0.43
≥ 350 FPL					0.52	0.28	0.97	0.04	0.58	0.29	1.18	0.13
Health insurance					0.92	0.32	2.64	0.88	0.88	0.31	2.48	0.80
High blood pressure					1.51	1.03	2.21	0.04	1.48	1.01	2.17	0.04
Diabetes					1.61	0.91	2.83	0.10	1.59	0.93	2.71	0.09
CVD					1.16	0.71	1.87	0.55	1.17	0.72	1.89	0.52
Cigarettes smoking (vs. Current smokers)												
Former smokers					0.43	0.20	0.91	0.03	0.45	0.21	0.95	0.04
Never smoked					0.77	0.37	1.63	0.49	0.81	0.38	1.73	0.58
Dental visits in the past year (vs. no annual dental visits)									0.92	0.56	1.52	0.73
Mouthwash in the last 7 days (vs. 0 days)												0.36
1–3									1.56	0.88	2.76	0.12
4–6									0.61	0.18	2.09	0.42
7									1.29	0.81	2.07	0.28
Dental flossing in the last 7 days (vs. 0 days)												0.02
1–3									0.41	0.19	0.89	0.03
4–6									0.91	0.37	2.22	0.83
7									0.51	0.28	0.91	0.03

*Note:* CVD: cardiovascular disease.

Abbreviations: AOR = adjusted odds ratio, FPL = federal poverty level, OR = Odds ratio.

Table 3 presents the logistic regression model results for the three domains of cognitive performance: low word memory, low AFT, and low DSST. Overall, the results are similar to those presented in Table 2. In Model 1, STL was statistically significant for low memory (OR = 2.81, 95% CI: 1.99–3.95), low AFT (OR = 4.88, 95% CI: 2.88–8.26), and low DSST (OR = 9.28, 95% CI: 4.68–18.39), indicating that STL was associated with higher odds of lower cognitive performance across the three domains. In Model 2, adjusting for other variables, STL remained statistically significant for low AFT (AOR = 2.14, 95% CI: 1.24–3.71), whereas the association with low DSST was not statistically significant (AOR = 2.03, *p* = 0.07).

**TABLE 3:** Logistic regression results by different cognitive domains.

Variables	Model 1				Model 2				Model 3			
	OR	95% CI		*p*	AOR	95% CI		*p*	AOR	95% CI		*p*
*DV = low word memory*												
Missing teeth (vs. 0 missing teeth)				< 0.001				0.05				0.11
1–7	1.35	0.97	1.89	0.07	0.68	0.46	1.00	0.05	0.69	0.46	1.03	0.07
8–27	2.81	1.99	3.95	< 0.001	1.05	0.69	1.60	0.80	1.00	0.65	1.53	0.98
Dental visits in the past year (vs. no annual dental visits)									0.97	0.69	1.36	0.84
Mouth washing times in the last 7 days (vs. 0 days)												0.90
1–3									1.09	0.69	1.72	0.70
4–6									0.82	0.39	1.74	0.60
7									1.03	0.70	1.52	0.87
Dental flossing tines in the last 7 days (vs. 0 times)												0.05
1–3									0.80	0.50	1.30	0.36
4–6									1.24	0.76	2.02	0.38
7									0.69	0.49	0.98	0.04

*DV = low AFT*												
Missing teeth (vs. 0 missing teeth)				< 0.001				0.01				0.03
1–7	2.23	1.30	3.82	0.01	1.42	0.81	2.48	0.21	1.46	0.84	2.55	0.17
8–27 (significant tooth loss)	4.88	2.88	8.26	< 0.001	2.14	1.24	3.71	0.01	1.99	1.15	3.44	0.02
Dental visits in the past year (vs. no annual dental visits)									0.93	0.64	1.36	0.72
Mouthwash in the last 7 days (vs. 0 days)												0.99
1–3									1.00	0.71	1.40	0.99
4–6									1.06	0.64	1.77	0.81
7									1.06	0.67	1.70	0.79
Dental flossing in the last 7 days (vs. 0 days)												0.02
1–3									0.64	0.40	1.01	0.05
4–6									0.37	0.20	0.66	0.01
7									0.66	0.47	0.93	0.02

*DV = low DSST*												
Missing teeth (vs. 0 missing teeth)				< 0.001				0.85				0.74
1–7	2.84	1.39	5.78	0.01	1.03	0.48	2.21	0.94	1.05	0.49	2.22	0.90
8–27 (significant tooth loss)	9.28	4.68	18.39	< 0.001	2.03	0.94	4.41	0.07	1.83	0.81	4.09	0.14
Dental visits in the past year (vs. no annual dental visits)									0.77	0.50	1.19	0.23
Mouthwash in the last 7 days (vs. 0 days)												0.32
1–3									1.46	0.86	2.47	0.16
4–6									1.76	0.69	4.49	0.23
7									1.12	0.76	1.65	0.56
Dental flossing in the last 7 days (vs. 0 days)												0.10
1–3									0.48	0.27	0.84	0.01
4–6									0.64	0.25	1.65	0.34
7									0.61	0.35	1.05	0.08

*Note:* Model 1 was unadjusted. Model 2 adjusted for age, sex, race/ethnicity, marital status, education level, family income level, insurance, high blood pressure, diabetes, cardiovascular disease, and cigarette smoking status. Model 3 additionally adjusted for dental visits, mouthwash use, and dental flossing.

Abbreviations: AOR = adjusted odds ratio, DV = dependent variable, OR = odds ratio.

In Model 3, where dental visits, mouthwash, and dental flossing were further adjusted, STL was not statistically significant in the model for low word memory (AOR = 1.00, *p* = 0.98) or in the low DSST model (AOR = 1.83, *p* = 0.14), while it was still significant in the low AFT model (AOR = 1.99, 95% CI: 1.15–3.44) but the magnitude was reduced substantially. Similar to findings in Table 2, neither having a recent dental visit nor using mouthwash was statistically significant (all *p*s > 0.05) in the models for the different cognitive domains. Dental flossing was significantly associated with several cognitive outcomes. For instance, in the low word memory model, daily flossing was associated with lower odds (AOR = 0.69, 95% CI: 0.49–0.98) of low word memory. In the low AFT model, dental flossing was statistically significant (*p* = 0.02). Specifically, daily flossing (AOR = 0.66, 95% CI: 0.47–0.93), or flossing 4–6 days (AOR = 0.37, 95% CI: 0.20–0.66) in the last 7 days were associated with lower odds of low AFT. Finally, in the low DSST model, flossing 1–3 days (AOR = 0.48, 95% CI: 0.27–0.84) was significant and daily flossing (AOR = 0.61, *p* = 0.08) was not significant. Taken together, these findings indicate that adjustment for dental care utilization and oral hygiene behaviors attenuated the observed association between tooth loss and cognitive performance across multiple cognitive domains (Table 3).

*Additional analysis results*. Interaction terms between tooth loss and each dental behavior variable were tested in the fully adjusted models. None of the interaction terms was statistically significant (all *p*s > 0.05).

## 4. Discussion

In this study, we examined whether adjustment for dental care utilization and oral hygiene behaviors influenced the observed association between tooth loss and cognitive performance in community‐dwelling older adults. We found that overall, tooth loss was associated with lower cognitive performance, assessed by global cognitive performance scores and scores of different cognitive domains, and adjustment for dental care utilization and oral hygiene behaviors attenuated the observed association between tooth loss and cognitive performance.

Unlike prior studies [3, 8, 30] that mainly assessed the association between tooth loss and cognitive impairment or dementia, our research assessed a more nuanced outcome: lower cognitive performance. The construct of “global low cognition” defined in this analysis was intended to identify lower overall cognitive performance rather than a clinical diagnosis of cognitive impairment. Across multiple models, tooth loss was associated with a greater likelihood of lower cognitive performance, particularly low verbal fluency, which is consistent with previous studies reporting associations between poor oral health and cognitive decline or dementia [3, 5, 8].

Importantly, in NHANES, participants with more severe cognitive impairment who were unable to complete the cognitive tests did not have cognitive test scores and therefore were not included in the analytic sample. Thus, the study sample offered an opportunity to explore the association between tooth loss and cognitive performance among community‐dwelling older adults, despite the bidirectional relationship between these factors [31, 32]. Our findings are consistent with previous observational studies reporting an association between tooth loss and cognitive performance [3, 5]. An alternative explanation is reverse causation. Even subtle cognitive decline may impair an individual’s ability to maintain daily oral hygiene, adhere to regular dental flossing, or seek routine dental care, ultimately contributing to periodontal disease and tooth loss. Thus, poor oral health may be both a contributor to and a consequence of declining cognitive function. The cross‐sectional nature of NHANES does not allow these temporal pathways to be disentangled. Therefore, longitudinal studies are needed to clarify the temporal relationship between oral health and cognitive decline.

Although no causal relationship can be inferred from this cross‐sectional study, to our knowledge, this is among the first studies to examine how adjustment for dental care utilization and oral hygiene practices influences the observed association between tooth loss and cognitive performance. The magnitude of the associations between tooth loss and low cognitive performance was reduced across the sequentially adjusted models, with further attenuation after dental visits, mouthwash use, and dental flossing were added in Model 3. Among the dental care variables examined, dental flossing was associated with lower odds of poor cognitive performance across several cognitive domains. From a life‐course perspective, both poor oral health (e.g., tooth loss) and cognitive decline accumulate over time [33, 34]. Furthermore, dental flossers may differ from nonflossers in important ways—they may have higher oral health literacy and higher socioeconomic status, which may account for the findings in this study. Although daily flossing was associated with better cognitive performance on several cognitive outcomes, this finding should not be interpreted as evidence of a direct causal effect. Regular flossing may instead serve as a marker of greater adherence to preventive health behaviors and stronger socioeconomic resources. Although we adjusted for several sociodemographic characteristics, residual confounding by these and other unmeasured factors cannot be excluded.

The observed odds ratios were modest; however, even relatively small differences in cognitive performance may be clinically meaningful at the population level because oral hygiene behaviors are common, inexpensive, and potentially modifiable. Nevertheless, the effect sizes observed in this cross‐sectional study should not be interpreted as evidence that improving oral hygiene alone would substantially prevent cognitive decline. Confirmation from longitudinal studies and intervention trials is required.

We also evaluated whether dental care utilization and oral hygiene behaviors modified the association between tooth loss and cognitive performance by testing interaction terms. None of the interaction terms reached statistical significance. While adjustment for dental care utilization and oral hygiene behaviors attenuated the observed association between tooth loss and cognitive performance, our findings do not support a statistically significant interaction between tooth loss and dental behaviors.

Although our study did not investigate the underlying biological mechanisms linking tooth loss and cognitive performance, several potential mechanisms have been suggested in the literature, including impaired masticatory function [9], periodontal inflammation [6], compromised nutritional intake [35], and increased risk of social isolation [36]—all of which may contribute to cognitive decline. Chronic periodontal inflammation may increase circulating proinflammatory cytokines, leading to systemic inflammation, disruption of the blood–brain barrier, and activation of microglia, thereby promoting neuroinflammation. In addition, oral microbiome dysbiosis and translocation of periodontal pathogens, particularly *Porphyromonas gingivalis*, have been implicated in Alzheimer’s disease pathology through chronic inflammation and neurodegeneration. Reduced masticatory function resulting from tooth loss may reduce sensory stimulation of the hippocampus and other brain regions involved in memory [37]. Regular dental flossing has been shown to reduce dental plaque accumulation, gingival inflammation, and periodontal disease progression [38]. It has therefore been hypothesized that maintaining good oral hygiene may reduce systemic inflammatory burden and neuroinflammation, though these biological pathways remain incompletely understood and require further investigation [14, 39].

Contrary to expectations and prior studies [12, 15, 40], dental visits were not significantly associated with cognitive performance in our analysis. This discrepancy may be due to methodological differences. Prior studies using longitudinal data such as the Health and Retirement Study (HRS) assessed dental care utilization over time [12, 40]. In contrast, NHANES relies on cross‐sectional data. In the data used for this analysis, detailed information on dental care was not collected, including type of provider (e.g., general dentists vs. specialists) or type of procedure performed (e.g., preventive vs. treatment). As such, it is difficult to draw definitive conclusions about the association between dental care utilization and cognitive outcomes from the findings of this study. Given its role in maintaining oral health, regular access to dental services remains important and should be encouraged.

A recent review [41] found that while toothbrushing was consistently associated with reduced dementia risk, other oral hygiene practices—including flossing and mouthwash use—were not. Differences in study populations (e.g., nursing home vs. community‐dwelling), outcomes (dementia vs. lower cognitive performance), and study designs (longitudinal vs. cross‐sectional) make direct comparisons difficult. Our study adds to the literature by demonstrating an association between dental flossing and better cognitive performance, although longitudinal studies are needed to better understand the relationship between oral hygiene behaviors and cognitive trajectories over time.

### 4.1. Limitations

Strengths of this study include the use of a large, nationally representative sample of community‐dwelling older adults and the assessment of multiple cognitive domains. Several limitations should also be acknowledged. First, toothbrushing frequency was unavailable in NHANES 2011–2014. Second, oral health behaviors and dental care utilization were self‐reported and therefore subject to recall bias. In addition, dental flossing and mouthwash use were assessed based on behaviors during the previous 7 days and may not reflect participants’ long‐term oral hygiene practices. Third, the cross‐sectional design precludes causal inference and residual confounding from unmeasured factors cannot be excluded. Fourth, participants unable to complete the cognitive assessments, including those with more severe cognitive impairment, were excluded, which may limit generalizability. The complete‐case analysis also excluded participants with missing covariate data, which may have introduced selection bias if missingness was systematically associated with oral health or cognitive performance.

Fifth, given the multiple cognitive outcomes examined, the possibility of Type I error due to multiple comparisons cannot be excluded. Finally, although NHANES 2011–2014 provide the most recent nationally representative data including both cognitive assessments and oral health measures, updated datasets are needed to evaluate temporal trends.

## 5. Conclusions

In crude analyses, tooth loss was associated with poorer cognitive performance across multiple domains, though these associations were attenuated after adjustment for sociodemographic, health‐related, dental care, and oral hygiene factors. Dental flossing was associated with better performance on several cognitive outcomes, though the pattern varied by flossing frequency and cognitive domain. Given the cross‐sectional design, these findings represent associations rather than evidence of causality. Longitudinal studies are needed to clarify the temporal relationships among tooth loss, oral hygiene behaviors, and cognitive performance.

## Funding

This work was supported in part by the National Institutes of Health (NIH) grant R01AG089856.

## Ethics Statement

The data (US NHANES) used in this project are in the public domain. Our study was conducted in accordance with the Declaration of Helsinki. Our study was exempt from IRB review.

## Consent

The authors have nothing to report.

## Conflicts of Interest

The authors declare no conflicts of interest.

## Data Availability

The data used for this study are from the National Health and Nutrition Examination Survey (NHANES). NHANES data are in the public domain, available at https://wwwn.cdc.gov/nchs/nhanes/default.aspx.
